# Prediction of abdominal CT body composition parameters by thoracic measurements as a new approach to detect sarcopenia in a COVID-19 cohort

**DOI:** 10.1038/s41598-022-10266-0

**Published:** 2022-04-19

**Authors:** I. Molwitz, A. K. Ozga, L. Gerdes, A. Ungerer, D. Köhler, I. Ristow, M. Leiderer, G. Adam, J. Yamamura

**Affiliations:** 1 Department of Diagnostic and Interventional Radiology and Nuclear Medicine, University Medical Center Hamburg-Eppendorf, Martinistraße 52, 20246 Hamburg, Germany; 2 Institute of Medical Biometry and Epidemiology, University Medical Center Hamburg-Eppendorf, Martinistraße 52, 20246 Hamburg, Germany

**Keywords:** Medical research, Risk factors

## Abstract

As most COVID-19 patients only receive thoracic CT scans, but body composition, which is relevant to detect sarcopenia, is determined in abdominal scans, this study aimed to investigate the relationship between thoracic and abdominal CT body composition parameters in a cohort of COVID-19 patients. This retrospective study included n = 46 SARS-CoV-2-positive patients who received CT scans of the thorax and abdomen due to severe disease progression. The subcutaneous fat area (SF), the skeletal muscle area (SMA), and the muscle radiodensity attenuation (MRA) were measured at the level of the twelfth thoracic (T12) and the third lumbar (L3) vertebra. Necessity of invasive mechanical ventilation (IMV), length of stay, or time to death (TTD) were noted. For statistics correlation, multivariable linear, logistic, and Cox regression analyses were employed. Correlation was excellent for the SF (r = 0.96) between T12 and L3, and good for the respective SMA (r = 0.80) and MRA (r = 0.82) values. With adjustment (adj.) for sex, age, and body-mass-index the variability of SF (adj. r^2^ = 0.93; adj. mean difference = 1.24 [95% confidence interval (95% CI) 1.02–1.45]), of the SMA (adj. r^2^ = 0.76; 2.59 [95% CI 1.92–3.26]), and of the MRA (adj. r^2^ = 0.67; 0.67 [95% CI 0.45–0.88]) at L3 was well explained by the respective values at T12. There was no relevant influence of the SF, MRA, or SMA on the clinical outcome. If only thoracic CT scans are available, CT body composition values at T12 can be used to predict abdominal fat and muscle parameters, by which sarcopenia and obesity can be assessed.

## Introduction

Sarcopenia and sarcopenic obesity have a worldwide prevalence over 10%^1,2^ and are associated with longer hospitalization stays^3^ and mortality^4^. In COVID-19 patients, a loss of lean muscle mass over the disease period has been described^5^, and a better thoracic muscle quality has been shown to be a protective factor against hospitalization, invasive mechanical ventilation (IMV), and death^6^. Also, obesity has been found to be significantly associated with higher hospitalization rates, IMV, intensive care unit (ICU) admission, and death^7^, while visceral and muscular fat amounts were related to the probability of ICU admission^8–10^.

In cases of suspected sarcopenia (e.g., reduced muscle strength) it is recommended to measure muscle quality and quantity^11^. To this purpose magnetic resonance imaging (MRI) and computed tomography (CT), between which body compositions results have been found to agree well^12^, are considered the gold standard^11^. In CT scans, which severely ill patients frequently receive, body composition measurements at the height of the third lumbar vertebra (L3) best represent the whole-body fat and muscle mass^13^. CT body composition parameters at L3 have been found to be of predictive value in many entities including COVID-19^9,10,14^. However, most COVID-19 patients only receive chest X-rays or thoracic CT scans and while there are reviews with hundreds of studies on abdominal CT body composition measurements^15,16^ experience on thoracic CT body composition parameters is less profound, with especially few studies on thoracic CT body composition assessment in COVID-19^5,6,17^. Furthermore, no consensus exists on which thoracic muscle groups to employ and proposed thoracic cut-off values to assess sarcopenia vary^18–21^. To profit from the experience on abdominal CT body composition parameters and reliably assess sarcopenia in patients who only receive thoracic CT scans, prediction of abdominal parameters would thus be of interest.

Therefore, it was the purpose of this study to evaluate the predictability of abdominal body composition parameters by thoracic CT parameters in a cohort of COVID-19 patients.

## Methods

### Study population

This explorative retrospective study was approved by the local ethics board (Ärztekammer Hamburg, Germany) with a waiver of informed written consent (WF-024/21). Study protocols and procedures were conducted in compliance with the Declaration of Helsinki.

Inclusion criteria were a RT-PCR positive test of SARS-CoV-2 and a contrast-enhanced CT scan of the thorax and abdomen between May 2020 and December 2020 at our University Medical Hospital. If patients received several CT scans during their hospitalization, the first scan was used. Exclusion criteria were: (a) non-ICU patients to generate a more homogenous collective, and (b) non-contrast-enhanced examinations because the muscle radiodensity attenuation (MRA) would differ between contrast-enhanced and non-contrast-enhanced scans^22^, which biases comparability between patients if no dual-energy CT scans for contrast-agent independent muscle characterization are available^23^. Furthermore, patients were excluded if their CT scans showed (c) artifacts in the paravertebral muscle, e.g., due to osteosynthesis material, (d) did not include the whole abdominal muscle area, or (e) displayed an open abdomen, as all of this could hinder the determination of the skeletal muscle area (SMA) and MRA at L3. In total n = 46 patients were included.

### CT scan and analysis

CT (SOMATOM Force®, Siemens Healthineers, Germany) parameters were 100/Sn150 kV, pitch 0.5, collimation 0.6 mm, and reconstructed slice thickness 5 mm. Image acquisition started 80 s after injection of contrast agent (Iomeprol [Imeron 350 M, Bracco IMAGING, Italy]).

**Figure 1:**
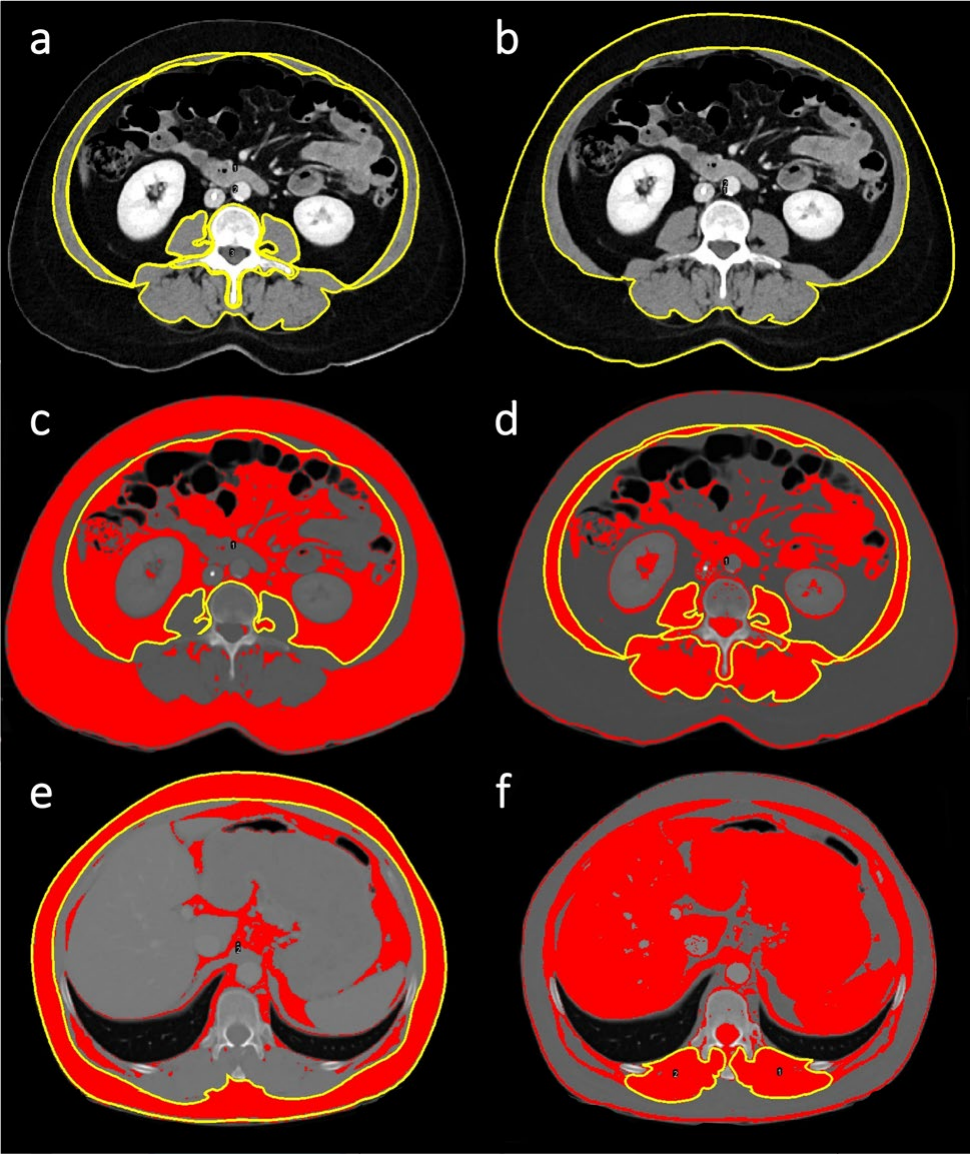
Regions of interest (ROI) for fat and muscle measurements on axial CT images. Measurements were performed on axial slices of contrast-enhanced CT scans of the thorax and abdomen at the level of the third lumbar vertebra (L3) (**a**–**d**) and the twelfth thoracic vertebra (T12) (**e**–**f**) with a muscle-specific (− 29 to + 150 Hounsfield units (HU)) and fat-specific threshold (− 190 to − 30 HU), respectively. Image (**a**) and (**b**) are pictured without the threshold to allow better visibility of anatomic structures. For the skeletal muscle area (SMA) at L3, the area of the ROI around L3 and of that around the abdominal muscles' inner circumference were subtracted from the area of the ROI along the outer muscles' circumference according to Gomez-Perez et al.^25^ (**a**). For the subcutaneous fat area at L3 and T12 the area of the ROI along the outer muscles' circumference was subtracted from the area of the ROI around the waist circumference (**b**, **e**). For the visceral fat area, a ROI was drawn along the inner circumference of the abdominal muscle (**c**). The circled muscles used to determine the muscle radiodensity attenuation (MRA) at L3 are shown in (**d**). For the SMA and MRA at T12, because of a potential measurement bias caused by the ribs, ROIs were defined along the posterior paraspinal muscles' circumference (**f**).

An axial CT slice at the mid-height of the 12th thoracic vertebra (T12) and L3, respectively, on which both transverse processes were equally depicted, was exported and further processed with the open-source software ImageJ (National Institutes of Health and the Laboratory for Optical and Computational Instrumentation, USA)^24^. At the level of L3, the abdominal muscles' outer and inner perimeter and the perimeter of L3 were manually contoured (Fig. 1a). The SMA was then determined by applying a muscle-specific threshold (− 29 to + 150 Hounsfield units (HU)) according to Gomez-Perez et al.^25^. The skeletal muscle index (SMI) was calculated from the SMA by correction for body size (SMA [cm^2^]/height^2^ [m^2^]). Additional regions of interest (ROI) were drawn along the waist circumference (WC) and around the whole abdominal muscles' circumference at L3. The subcutaneous fat area (SF) was calculated by subtracting the outer muscle area from the whole body area (WC ROI) after application of a fat-specific threshold (− 190 to − 30 HU) (Fig. 1b). The visceral fat area was given by the ROI along the abdominal muscles' inner perimeter and the fat-specific threshold (Fig. 1c). The MRA was determined from the whole abdominal muscle at L3 after application of the muscle-specific threshold (Fig. 1d) to avoid underestimation of myosteotosis which can occur if measurements are restricted to, e.g., the psoas muscle^26^. Whole-body fat mass [kg] and visceral fat mass [kg] were calculated as proposed by Zopf et al., who have proven good agreement to bioelectrical impedance analysis (BIA) body composition results^13^.

At the level of T12, ROIs were defined along the WC, the outer muscle perimeter, and the posterior paraspinal muscles' circumference. The SF area was given by subtraction of the outer muscle area from the whole-body area (WC ROI) and the fat-specific threshold (Fig. 1e). As the ribs could bias determination of the intercostal muscle area, the SMA and MRA at T12 were solely noted for the posterior paraspinal muscles (Fig. 1f). Finally, a recently proposed fat to muscle ratio (FMR) on thoracic CT scans of COVID-19 patients was calculated by: WC at T12/((circumference of the right posterior paraspinal muscle + circumference of the left posterior paraspinal muscle)/2)^17^.

### Clinical parameters and sarcopenia classification

The clinical parameters necessity of IMV, length of stay until hospital discharge, or time to death (censoring 15th February 2021) were noted. Patients' weight was classified according to the World Health Organization's (WHO) weight classes^27^. Patients were classified as sarcopenic based on their MRA using the sex, age, and body mass index (BMI) specific 5th percentile of van der Werf et al.^28^. As different SMI cut-offs have been suggested and are applied for Caucasian populations in the literature, SMI values were categorized as sarcopenic according to three widely used systems: the sex-specific cut-offs of Prado et al.^29^, the sex-specific cut-offs of Martin et al.^30^, which for men also consider the BMI, and the sex-, age-, and BMI-specific percentiles of van der Werf et al.^28^.

### Statistics

For patients' characteristics absolute and relative numbers for categorical data and mean with standard deviation or median, maximum, and minimum for continuous data were reported.

For correlation between the normally distributed variables at T12 and L3 (WC T12 to WC L3, SF T12 to SF L3, MRA T12 to MRA L3, SMA T12 to SMA L3), Pearson's correlation was applied. Agreement between the WC at T12 and L3, and the respective values of the SF, MRA, and SMA was evaluated by Bland–Altman analysis and the intraclass correlation coefficient (ICC). For predictability of the WC, SF, MRA, and SMA at L3 by the respective values on T12 and between the FMR at T12 and the SMA at L3 multivariable linear regression analyses were employed. To investigate the relationship between CT measurements and the metric outcome variable “length of stay” (skewed data distribution) Spearman's correlation was used. For the CT measurements' relation to the nominal clinical outcome variable “IMV” multivariable logistic regression analysis was employed, for the “time to death” Cox regression was used. All regression analyses with CT measurements as independent variables were adjusted (adj.) for sex, age, and BMI.

Multivariable logistic regression was applied to investigate the relationship between the sarcopenia classification results of Prado et al., Martin et al., and van der Werf et al. and the outcome variable “IMV”. For evaluation of the influence of the sarcopenia classification results on “time to death” a Cox regression was applied. Multivariable linear regression was used to determine the relation of the sarcopenia classification results to the logarithmized variable “length of stay”. Because the sarcopenia classification systems in part already include sex, age, and BMI, these analyses were not adj. for sex, age, or BMI.

Because of the explorative study design no post-hoc power analysis was performed^31^, all given p-value are thus descriptive, and no adjustment for multiple testing was conducted.

## Results

### Study population

Of 46 included patients 19 (41.3%) were female and 27 (58.7%) were male. Mean age was 64.4 years with a standard deviation (SD) of ± 11.4, a minimum of 29 years, and a maximum of 84 years. Mean BMI was 27.3 kg/m^2^ ± 6.2 SD. According to the WHO's classification, most patients were classified as obese grade I (≥ 25 kg/m^2^)^27^. Male patients had a slightly lower mean BMI with a smaller SD (26.2 kg/m^2^ ± 5.6) than women (29.2 kg/m^2^ ± 6.9). Of 39 (84.8%) patients who required IMV, 19 were female (48.7% out of 39) and 27 male (69.2% out of 39). The median length of stay at ICU was 23 days, with 15 days for female and 29 days for male patients. Variance was large with a one-day minimum and 233 days maximum length of stay. At the end of study, 26 patients (56.5%), of which 8 were female (30.8% out of 26) and 18 were male (69.2% out of 26), were deceased. 17 patients (37.0%) were discharged.

Most common comorbidities were arterial hypertension (n = 25, 54.3%) and type II diabetes (n = 23, 50.0%), followed by cardiovascular diseases, e.g., post myocardial infarction, cardiomyopathy, coronary heart disease (n = 16, 34.8%), cancer -mostly leukemia and lymphoma- (n = 12, 26.1%), and renal failure (n = 7, 15.2%). 22 (47.8%) patients were dependent on intermittent or continuous dialysis and 13 (28.3%) patients received extracorporeal membrane oxygenation (ECMO) therapy.

### Distribution of CT body composition parameters and sarcopenia

Sex-specific mean values and SD of all CT parameters, the SMI, BMI, visceral and body fat mass are provided in Table 1.

**Table 1:** Sex-specific muscle and fat parameters.

	Female (n = 19)		Male (n = 27)	
	Mean	SD	Mean	SD
SMA L3 [cm^2^]	96.49	23.23	125.37	34.99
SMI	35.68	9.68	39.84	10.25
SMA T12 [cm^2^]	28.72	9.19	32.07	10.22
MRA L3 [HU]	32.1	11.7	38.6	12.6
MRA T12 [HU]	33.8	14.2	39.2	15.8
SUBCUTANEOUS FAT L3 [cm^2^]	303.26	130.87	178.66	121.28
SUBCUTANEOUS FAT T12 [cm^2^]	195.80	105.28	102.27	84.14
VISCERAL FAT AREA L3 [cm^2^]	195.83	111.57	233.42	101.54
VISCERAL FAT MASS [kg]	3.11	1.53	1.62	1.36
WHOLE BODY FAT MASS [kg]	38.81	12.23	24.59	13.85
BMI [kg/m^2^]	29.3	6.9	25.7	5.3

Sex-specific mean and standard deviation (SD) of the skeletal muscle area (SMA), muscle radiodensity attenuation (MRA), and subcutaneous fat area are provided at the level of the third lumbar vertebra (L3) and twelfth thoracic vertebra (T12) on axial contrast-enhanced computed tomography slices. Additionally, mean and SD of the visceral fat area at L3, visceral and whole-body fat mass, the skeletal muscle index (SMI), and the body mass index (BMI) are listed.

**Figure 2:**
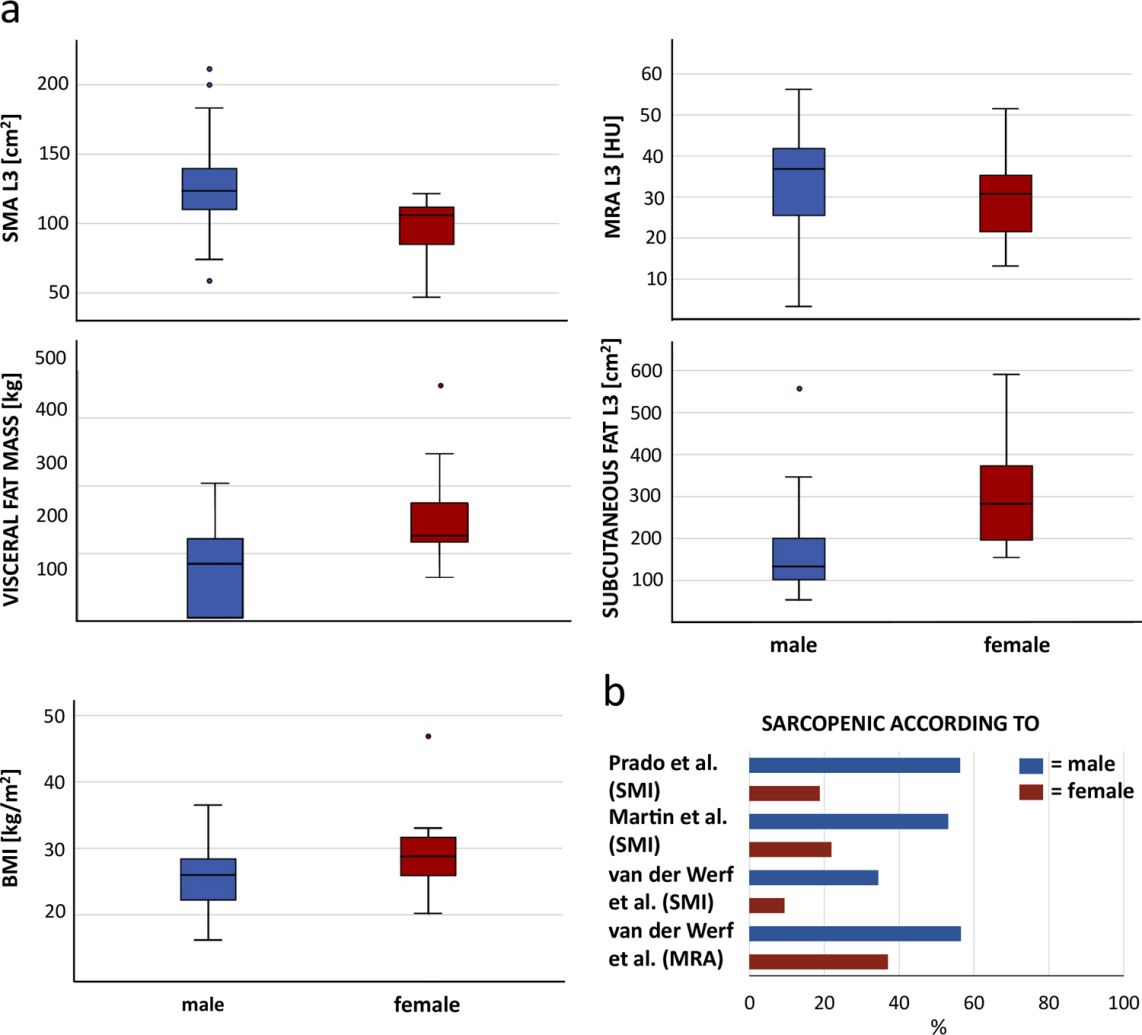
Sex-specific distribution of fat and muscle parameters at L3 and percentages of sarcopenic patients. Distribution of the skeletal muscle area (SMA), muscle radiodensity attenuation (MRA), and the subcutaneous fat area at the level of the third lumbar vertebra (L3), the visceral fat mass, and the body mass index (BMI) in men (blue) and women (red) (**a**). Male patients showed a lower mean BMI, less subcutaneous fat, and a lower visceral fat mass. The SMA and MRA were higher in men, however, according to all employed sarcopenia cut-off values, men were more often classified as sarcopenic than women (**b**).

In men, MRA values at T12 and L3 were slightly higher (T12: 39.2 HU; L3: 33.8 HU), which indicated less fat accumulation within the muscle fibers, than in women (38.6 HU; 32.1 HU). However, compared to the sex-, age-, and BMI-specific percentiles of van der Werf et al. for the MRA at L3, more men were below the 5th percentile (n = 26, 56.5%) and thus considered to have low muscle quality, than women (n = 17, 37.0%). Like the BMI, the subcutaneous fat area, the visceral fat mass, and whole-body fat mass were smaller in male patients (Table 1). The SMI could be determined for 20 male and 12 female patients, for whom body height was given. Judged by the sarcopenia cut-off values, men in this study collective were sarcopenic up to two times more often than women. In detail: according to the cut-off values for the SMI of Prado et al.^29^ 18 men and six women were sarcopenic, by the cut-off for the SMI of Martin et al.^30^ 17 men and seven women. 11 male patients and three female patients were below the sex-, age-, and BMI-specific 5th percentile for the SMI proposed by van der Werf et al.^28^, while five male and three female patients were below the 5th percentile for the MRA. An overview of the sex-specific distribution of sarcopenia classification results and variance of fat as well as muscle parameters at L3 is provided in Fig. 2.

### Relationship between CT parameters at T12 and L3

Correlation of the WC (r = 0.93), SF (r = 0.96), MRA (r = 0.82), and the SMA (r = 0.80) between T12 and L3 was strong. Agreement between WC values at T12 and L3 (ICC: 0.901 [95% confidence interval (95% CI) 0.778–0.951]) was good with a mean difference of 2.86 cm [95% CI − 7.64 to 13.35]. For the MRA of the paraspinal muscle at T12 and the whole abdominal muscle at L3 agreement was moderate (0.750 [95% CI 0.412–0.882]) with a mean difference of − 5.8 HU [95% CI − 22.3 to 10.7]. The 95% CI of the ICC for the SF at T12 and L3 was large (0.722 [95% CI − 0.070 to 0.917]), as was the mean difference with 85.55 cm^2^ [95% CI − 8.58 to 179.68]. As expected, the paraspinal muscle area at T12 differed from that of the whole abdominal muscle at L3 (ICC: 0.067 [95% CI − 0.043 to 0.254]) with a mean difference of 90.52 cm^2^ [95% CI 27.41–153.63].

**Figure 3:**
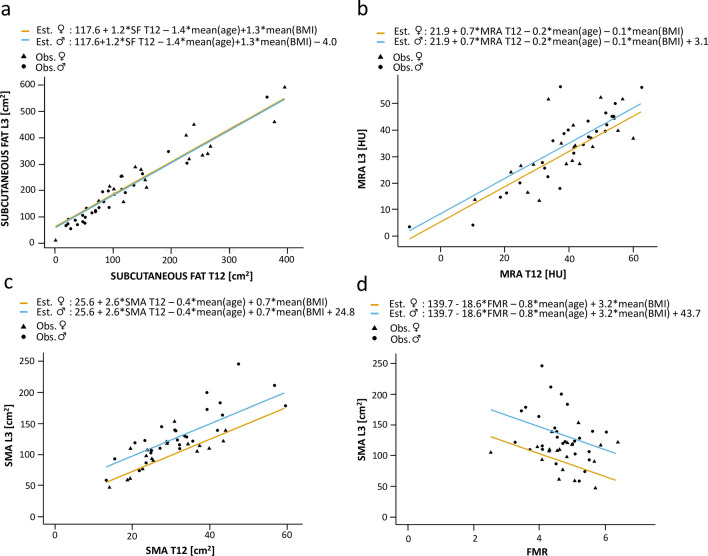
Sex-specific estimated regression lines. Results of multivariable linear regression analyses with the adjusting variables sex, age, and body mass index (BMI). The respective independent variables were subcutaneous fat (SF) at the twelfth thoracic vertebra (T12) (**a**), the muscle radiodensity attenuation (MRA) at T12 (**b**), the skeletal muscle area (SMA) at T12 (**c**), and the fat to muscle ratio (FMR) at T12 (**d**). Respective dependent variables were subcutaneous fat at the level of the third lumbar vertebra (L3) (**a**), the muscle radiodensity attenuation (MRA) at L3 (**b**), and the skeletal muscle area (SMA) at L3 (**c**, **d**). Est. = estimated, Obs. = observed.

Concerning the predictability of the abdominal body compositions parameters by thoracic values, with adjustment for sex, age, and BMI, the SF area at T12 explained the SF area's variation at L3 well (adj. r^2^ = 0.93) (Table 2, Fig. 3). An increase of the SF area at T12 by 1 cm^2^ was mirrored by an adj. mean increase of the SF area at L3 by 1.24 cm^2^ (95% CI 1.02–1.45) (Table 2). Comparably, results were good for the MRA (adj. r^2^ = 0.67): an increase of 1 HU at T12 was mirrored by an adj. mean increase of 0.67 HU (95% CI 0.45–0.88) at L3 (Fig. 3). The model also fitted well for the paraspinal muscle area at T12 and the whole abdominal muscle area (SMA) at L3 (adj. r^2^ = 0.76; adj. mean difference = 2.59 [95% CI 1.92–3.26]).

**Table 2:** Multivariable linear regression results of CT parameters.

	Corrected r^2^	Constant	Adj. mean difference	95% CI	P-value
**SUBCUTANEOUS FAT L3**					
**SUBCUTANEOUS FAT T12**	**0.93**	**117.61**	**1.24**	**1.02** to **1.45**	**< 0.001**
Sex			− 4.0	− 34.93 to 26.93	0.793
Age			− 1.43	− 2.80 to − 0.05	0.043
BMI			1.25	− 1.95 to 4.45	0.430
**MRA L3**					
**MRA T12**	**0.67**	**21.94**	**0.67**	**0.45** to **0.88**	**< 0.001**
Sex			3.11	− 2.99 to 9.21	0.305
Age			− 0.22	− 0.56 to 0.11	0.182
BMI			− 0.09	− 0.58 to 0.40	0.714
**SMA L3**					
**SMA T12**	**0.76**	**25.64**	**2.59**	**1.92** to **3.26**	**< 0.001**
Sex			24.79	10.22 to 39.35	0.002
Age			− 0.36	− 1.06 to 0.34	0.301
BMI			0.69	− 0.48 to 1.86	0.235
**FMR**	**0.35**	**139.70**	− **18.63**	− **35.08** to − **2.18**	**0.028**
Sex			43.65	20.13 to 67.18	0.001
Age			− 0.78	− 1.93 to 0.36	0.172
BMI			3.25	1.02 to 5.47	0.006
**SMI**					
**SMA T12**	**0.64**	**7.41**	**0.80**	**0.56** to **1.03**	**< 0.001**
Sex			2.35	− 2.51 to 7.21	0.330
Age			− 0.03	− 0.27 to 0.20	0.785
BMI			0.26	− 0.14 to 0.65	0.199
**FMR**	**0.11**	**41.58**	− **5.14**	− **10.50** to **0.22**	**0.059**
Sex			7.25	− 0.36 to 14.85	0.061
Age			− 0.13	− 0.49 to 0.24	0.488
BMI			0.90	0.14 to 1.66	0.023

Results of the multivariable linear regression for CT parameters at T12 as independent variable and CT parameters at L3 as dependent variable (in bold letters) including the results of the adjusting variables sex, age, and body mass index (BMI) (not bold).

Adj. mean difference = adjusted mean difference, CI = confidence interval, L3 = at the level of the third lumbar vertebra, T12 = at the level of the twelfth thoracic vertebra, MRA = muscle radiodensity attenuation in Hounsfield units, SMA = skeletal muscle area [cm^2^], FMR = fat to muscle ratio calculated by waist circumference at the height of T12/((circumference of the right posterior paraspinal muscle + circumference of the left posterior paraspinal muscle)/2) according to Kottlors et al.^17^.

With adjustment for sex, age, and BMI, less variation of the SMA at L3 or the SMI was explained by the FMR (adj. r^2^ = 0.35; adj. r^2^ = 0.11). An increase of the FMR by one point, which can be caused by an increase of the WC or decrease of the posterior paraspinal muscle circumference, was associated with an adj. mean decrease of the SMA of 18.63 cm^2^ with a large confidence interval (95% CI − 35.08 to − 2.18) (Table 2, Fig. 3).

### Association between CT parameters and the clinical outcome

No relationship between the SMA, the SMI, the MRA, the subcutaneous or visceral fat area, the visceral or total body fat mass, the FMR, and the clinical outcome parameters length of stay, IMV, or time to death was found (Table 3). Similarly, there was no relevant association between patients who were classified as sarcopenic by the cut-off-based systems of Prado et al., Martin et al., or van der Werf et al. and length of stay, IMV, or time to death (Table 3).

**Table 3:** Relationship between body composition parameters, sarcopenia classification results, and the clinical outcome parameters length of stay, invasive mechanical ventilation (IVM), and time to death.

	Spearman's correlation		Logistic regression		Cox regression	
	Length of stay		IMV		Time to death	
	r		Adj. OR [95% CI]	P-value	Adj. HR [95% CI]	P-value
SMA L3 [cm^2^]	− 0.211		0.996 [0.961–1.033]	0.837	1.002 [0.986–1.018]	0.841
SMI	− 0.265		1.025 [0.913–1.150]	0.677	1.008 [0.958–1.060]	0.762
SMA T12 [cm^2^]	− 0.178		1.019 [0.918–1.130]	0.724	0.985 [0.941–1.031]	0.513
MRA L3 [HU]	− 0.186		1.053 [0.955–1.161]	0.299	1.007 [0.959–1.057]	0.792
MRA T12 [HU]	− 0.023		0.998 [0.923–1.078]	0.956	0.996 [0.963–1.030]	0.807
SUBCUTANEOUS FAT L3 [cm^2^]	− 0.236		0.983 [0.963–1.003]	0.093	1.001 [0.995–1.007]	0.683
SUBCUTANEOUS FAT T12 [cm^2^]	− 0.227		0.981 [0.954–1.009]	0.190	0.998 [0.989–1.007]	0.653
VISCERAL FAT AREA L3 [cm^2^]	0.027		1.005 [0.991–1.020]	0.500	0.997 [0.990–1.005]	0.495
VISCERAL FAT MASS [kg]	− 0.356		0.342 [0.070–1.676]	0.186	1.225 [0.752–1.996]	0.415
WHOLE BODY FAT MASS [kg]	− 0.349		0.907 [0.781–1.053]	0.198	1.008 [0.960–1.059]	0.736
FMR	− 0.025		0.213 [0.035–1.276]	0.090	1.492 [0.567–3.931]	0.418

	Linear regression		OR [95% CI]	P-value	HR [95% CI]	P-value
	Length of stay					
	Mean diff. [95% CI]	P-value				
Sarcopenic patients according to Prado et al.	− 0.155 [− 0.477 to 0.167]	0.333	4.200 [0.645 to 27.362]	0.133	0.535 [0.122 to 2.345]	0.407
Sarcopenic patients according to Martin et al.	− 0.098 [− 0.423 to 0.227]	0.543	1.667 [0.243 to 11.448]	0.603	0.435 [0.100 to 1.900]	0.268
Patients < 5th SMI percentile of van der Werf et al.	− 0.131 [− 0.413 to 0.150]	0.348	1.714 [0.266 to 11.060]	0.571	0.870 [0.343 to 2.209]	0.770
Patients < 5th MRA percentile of van der Werf et al.	− 0.180 [− 0.500 to 0.140]	0.259	–*	–*	1.170 [0.414 to 3.305]	0.768

Regression analyses for the metric independent variables were adjusted for sex, age, and body mass index (BMI). Detailed results for all adjusting variables are listed in Supplement Table 1.

IMV = invasive mechanical ventilation, SMA = skeletal muscle area, L3 = at the level of the third lumbar vertebra, SMI = skeletal muscle index, T12 = at the level of the twelfth thoracic vertebra, MRA = muscle radiodensity attenuation, HU = Hounsfield units, FMR = fat to muscle ratio, adj. OR = adjusted odds ratio, CI = confidence interval, Adj. mean difference = adjusted mean difference, Adj. HR = adjusted hazard ratio.

*Model not applicable due to low event numbers.

The correlation and regression results for metric CT body composition parameters and the cut-off-based sarcopenia classification systems to length of stay, IMV, and time to death are listed in Table 3. The corresponding results of the adjusting variables are additionally provided in the Supplement Table 1.

## Discussion

The main findings of this study are that fat and muscle parameters measured on axial CT slices at the level of T12 and L3 correlate well and that in patients who only receive thoracic CT scans, like most COVID-19 patients, T12 values can be used to predict L3 values, for which long-term experience in determining sarcopenia and obesity exists.

The importance of CT body composition measurements is due to the fact that in contrast to two-dimensional techniques as dual-energy X-ray absorptiometry, no assumptions on the distribution of muscle and fat compartments are necessary^32^ and opposed to BIA CT results are less influenced by the patient's hydrational status^33^. Of the multiple studies and reviews which have been published on CT body composition^34–36^, studies which investigated fat or muscle parameters at other heights than L3 are the minority. Concerning other abdominal levels, e.g., only one out 20 studies in a review addressing body composition measurements in cancer^34^, and 18 out of 177 studies in a review on the MRA^36^ were not carried out at the level of L3. This may be explicable by the fact that the highest correlation of the skeletal muscle area to the whole-body muscle volume was described for analyses in the region of L3^37,38^. Similarly, compared to number of studies at the level of L3 there are few studies on thoracic CT body composition measurements, e.g., in patients with lung cancer^39,40^, or aortic valve repairment^20,21^. In COVID-19, studies on thoracic body composition measurements have been carried out at the height of the 7th and 8th thoracic vertebra^6^, at T12^5,17^, and for different muscle groups like the pectoralis muscle^6^ or the autochthones spine muscle^5,17^. The variation of measurement levels, of employed muscle groups, and the difference between proposed thoracic cut-off values for sarcopenia detection^19–21^, however, illustrate the lack of experience with thoracic CT body composition measurements. This supports the alternative approach, pursued by this study, of using thoracic measurements to predict abdominal body composition values, for which interpretation considerably more experience exists.

When comparing this study’s results to the literature, correlation results (SMA r = 0.80, MRA r = 0.82) are comparable to that of Derstine et al., who described moderate to excellent agreement for the correlation between all levels from T10 to L5 (SMA r = 0.65–0.95, MRA r = 0.63–0.95)^19^. As the SMI (SMA/height^2^) is regularly based on measurements at the L3 landmark^11^, it was not calculated for T12 in this study. Instead, the correlation for the SMA at T12 and L3 (r = 0.8) was investigated, which was in the same range as the results of Nemec et al., who determined and correlated the SMI at both levels (r = 0.71)^20^.

Regarding agreement between values at T12 and L3, only the WC showed good agreement, while for the MRA agreement was moderate. This is in accordance with Derstine et al. who described a significant variance of MRA values between T11 and L5 and 17 other thoracic and lumbar vertebrae pairings. It can thus be argued that MRA differences between the variable measurement heights are at least likely and thoracic MRA values cannot be simply considered representative for L3 values. Instead, the exact relation between T12 and L3 values as investigated in this study needs to be known.

Concerning regression results, compared to the results of Nemec et al., who modeled the SMI at T12 (men r^2^ = 0.546, women r^2^ = 0.477) by L3 SMI values^20^, the model for the SMA at L3 by T12 values in this study explained more of the variance (r^2^ = 0.76). Similarly, more of the L3 variance for both the SMA (r^2^ = 0.76) and MRA (r^2^ = 0.67) was explained in this study, than by a model of the SMI calculated for the 4th thoracic vertebra (T4) and L3 (male: r^2^ = 0.5, female r^2^ = 0.28) or the MRA at T4 and L3 (male: r^2^ = 0.50, female: r^2^ = 0.58)^41^. Compared to a study which used multivariable regression analysis to predict L3 values by the cervical muscle area (r = 0.785), this study’s result for the SMA (r = 0.76) are in good agreement.

That male patients were more often classified as sarcopenic in this study, is in accordance with previous findings. For example, Iannuzzi-Sucich et al. who assessed sarcopenia by BIA and clinical testing in a healthy older population, found men to be sarcopenic 4% more often than women, a discrepancy which increased in the age cohort of those over 80 years^42^. Similarly, higher rates of sarcopenic male patients have been described in some CT based body composition studies^43,44^. However, it should be noted that contradictory study results with a slightly higher sarcopenia prevalence among women have also been published^45^ and that the patient number of this study’s collective was limited.

Surprisingly, nearly no association was found between body composition parameters and the outcome variables length of stay, IMV, or time to death. Besides the overall patient number, this might be due to the study collective which consisted of already severely ill ICU patients, who furthermore had variable primary conditions (e.g., leukemia, cardiac, or renal primary conditions). Moreover, the predefined inclusion criterium of a CT scan of the thorax and abdomen, which in COVID-19 patients is only performed if an additional abdominal issue, e.g., due to rising lactate is suspected or for vascular imaging before installation of an ECMO therapy, caused a further selection of extremely ill patients. It is likely that in such study collective body composition parameters have less influence on the clinical outcome than other influencing factors as, e.g., bacterial superinfection, thrombosis, embolism, or bleeding. However, the negative impact of sarcopenia on the clinical outcome in the healthy older population^11,46,47^ is known, and effects of obesity^7,8^, reduced muscle mass^10^, or muscle quality^6^ on COVID-19 patients have been described. Also, while no association for the FMR to IMV or time to death was found, Kottlors et al. have described its use to predict ICU admission^17^. Determination of muscle and fat parameters at L3 to detect sarcopenia, obesity, or sarcopenic obesity more reliably than by the body weight or BMI, is thus nevertheless relevant to ensure best nutritional support and physical therapy in COVID-19-patients.

Limitations of this study are the heterogeneity of the study collective, the missing body size in 14 patients which reduced group size for the SMI analysis and thus SMI-based sarcopenia classification, and the overall patient number. This is because COVID-19 patients beside chest X-rays mostly receive thoracic CT scans. The number of patients, who, due to severe disease progression or complications, needed CT scans of the thorax and abdomen was thus limited. However, all patients who received CT scans of the thorax and abdomen during the study period were included. The restricted number of COVID-19 patients with CT scans of the thorax and abdomen also highlights the relevance of this study: to evaluate the predictability of established abdominal fat and muscle parameters by thoracic values in patients who only receive thoracic CT scans.

Concerning future studies, it would be interesting to apply this study’s results to less seriously ill COVID-19 patients with thoracic CT scans, to better evaluate the relation of the predicted abdominal CT values to the clinical outcome. In this context care should be taken to not only employ cut-off-based sarcopenia classification systems, which inherently results in a loss of information but as done in this study additionally perform analyses of the metric measurement data. Finally, to improve measurement speed and prospectively include CT body composition measurements into clinical routine the use of (semi)automated body composition analyzes as evaluated for abdominal CT slices should be investigated for thoracic scans as well^48^.

## Conclusion

This study demonstrates that CT body composition parameters at L3 may be predicted by fat and muscle measurements at T12. In patients who only receive thoracic CT scans, as most COVID-19 patients, conclusions on abdominal CT body composition parameters, for which long-term experience in the detection of sarcopenia and obesity exists, can thus be derived.

## Supplementary Information

Supplementary Table 1.

## Data Availability

The datasets generated during and/or analysed during the current study are available from the corresponding author on reasonable request.
