# Supplementary material for: Prediction of abdominal CT body composition parameters by thoracic measurements as a new approach to detect sarcopenia in a COVID-19 cohort
Source: Sci Rep. 2022 Apr 19;12:6443. doi: 10.1038/s41598-022-10266-0 (PMC9017415; doi:10.1038/s41598-022-10266-0)
Supplement: Supplementary file 1 — Supplementary Table 1. [file 41598_2022_10266_MOESM1_ESM.docx]

Prediction of abdominal CT body composition parameters by thoracic measurements as a new approach to detect sarcopenia in a COVID-19 cohort

Molwitz I^1*^, Ozga AK^2^, Gerdes L^1^, Ungerer A^1^, Köhler D^1^, Ristow I^1^, Leiderer M^1^, Adam G^1^, Yamamura J^1^

*Affiliations*

^1^Department of Diagnostic and Interventional Radiology and Nuclear Medicine, University Medical Center Hamburg-Eppendorf, Martinistraße 52, 20246 Hamburg, Germany

^2^Institute of Medical Biometry and Epidemiology, University Medical Center Hamburg-Eppendorf, Martinistraße 52, 20246 Hamburg, Germany

*Corresponding author

Dr. med. Isabel Molwitz MD, ORCID-ID: 0000-0003-4542-0140, i.molwitz@uke.de, phone: +49 152 22 81 70 04, fax: +49 40 7410 54640, Department of Diagnostic and Interventional Radiology and Nuclear Medicine, University Medical Center Hamburg-Eppendorf, Martinistraße 52, 20246 Hamburg, Germany

Ann-Kathrin Ozga, PhD, a.ozga@uke.de, Institute of Medical Biometry and Epidemiology, University Medical Center Hamburg-Eppendorf, Martinistraße 52, 20246 Hamburg, Germany

Laura Gerdes, laura.gerdes@ymail.com, Department of Diagnostic and Interventional Radiology and Nuclear Medicine, University Medical Center Hamburg-Eppendorf, Martinistraße 52, 20246 Hamburg, Germany

Arthur Ungerer, ungerer.a@gmx.de, Department of Diagnostic and Interventional Radiology and Nuclear Medicine, University Medical Center Hamburg-Eppendorf, Martinistraße 52, 20246 Hamburg, Germany

Dr. med. Daniel Köhler, MD, daniel.koehler@uke.de, Department of Diagnostic and Interventional Radiology and Nuclear Medicine, University Medical Center Hamburg-Eppendorf, Martinistraße 52, 20246 Hamburg, Germany

Dr. med. Inka Ristow, MD, i.ristow@uke.de, Department of Diagnostic and Interventional Radiology and Nuclear Medicine, University Medical Center Hamburg-Eppendorf, Martinistraße 52, 20246 Hamburg, Germany

Dr. med. Miriam Leiderer, MD, m.leiderer@uke.de, Department of Diagnostic and Interventional Radiology and Nuclear Medicine, University Medical Center Hamburg-Eppendorf, Martinistraße 52, 20246 Hamburg, Germany

Professor Dr. med. Gerhard Adam, MD, g.adam@uke.de, Department of Diagnostic and Interventional Radiology and Nuclear Medicine, University Medical Center Hamburg-Eppendorf, Martinistraße 52, 20246 Hamburg, Germany

Professor Dr. med. Jin Yamamura, MD, j.yamamura@uke.de, Department of Diagnostic and Interventional Radiology and Nuclear Medicine, University Medical Center Hamburg-Eppendorf, Martinistraße 52, 20246 Hamburg, Germany

*__________________________________________________________________________________*

*Declaration* *of interest*

The authors declare that they have no conflict of interest.

***Supplement, Table 1*** *Relationship between CT body composition parameters and the clinical outcome parameters invasive mechanical ventilation (IVM) and time to death including results for the adjusting variables sex, age, and body mass index (BMI).*

|  | Logistic Regression | | Cox Regression | |
| --- | --- | --- | --- | --- |
|  | **IMV** | | **Time to death** | |
|  | Adj. OR [95%CI] | P-Value | Adj. HR [95%CI] | P-Value |
| **SMA L3 [cm2]** | 0.996 [0.961-1.033] | 0.837 | 1.002 [0.986-1.018] | 0.841 |
| **Sex** | 0.134 [0.009-2.029] | 0.147 | 1.688 [0.422-6.743] | 0.459 |
| **Age** | 0.918 [0.814-1.035] | 0.162 | 0.993 [0.939-1.050] | 0.812 |
| **BMI** | 0.942 [0.784-1.131] | 0.520 | 1.006 [0.927-1.092] | 0.886 |
| **SMI** | 1.025 [0.913-1.150] | 0.677 | 1.008 [0.958-1.060] | 0.762 |
| **Sex** | 0.098 [0.008-1.249] | 0.074 | 1.831 [0.497-6.741] | 0.363 |
| **Age** | 0.915 [0.809-1.035] | 0.157 | 0.991 [0.941-1.044] | 0.739 |
| **BMI** | 0.933 [0.773-1.124] | 0.464 | 1.012 [0.933-1.099] | 0.766 |
| **SMA T12 [cm2]** | 1.019 [0.918-1.130] | 0.724 | 0.985 [0.941-1.031] | 0.513 |
| **Sex** | 0.092 [0.007-1.271] | 0.075 | 1.963 [0.577-6.677] | 0.280 |
| **Age** | 0.915 [0.808-1.037] | 0.164 | 0.983 [0.932-1.038] | 0.539 |
| **BMI** | 0.929 [0.774-1.115] | 0.427 | 1.010 [0.929-1.08] | 0.812 |
| **MRA L3 [HU]** | 1.053 [0.955-1.161] | 0.299 | 1.007 [0.959-1.057] | 0.792 |
| **Sex** | 0.087 [0.008-.991] | 0.049 | 1.781 [0.521-6.094] | 0.358 |
| **Age** | 0.936 [0.822-1.065] | 0.314 | 0.997 [0.932-1.066] | 0.924 |
| **BMI** | 0.940 [0.777-1.137] | 0.525 | 1.012 [0.927-1.104] | 0.791 |
| **MRA T12 [HU]** | 0.998 [0.923-1.078] | 0.956 | 0.996 [0.963-1.030] | 0.807 |
| **Sex** | 0.114 [0.012-1.094] | 0.060 | 1.783 [0.525-6.057] | 0.354 |
| **Age** | 0.917 [0.809-1.038] | 0.171 | 0.986 [0.927-1.050] | 0.664 |
| **BMI** | 0.936 [0.783-1.118] | 0.467 | 1.005 [0.923-1.094] | 0.910 |
| **SUBCUTANEOUS FAT L3 [cm2]** | 0.983 [0.963-1.003] | 0.093 | 1.001 [0.995-1.007] | 0.683 |
| **Sex** | 0.017 [0.001-0.565] | 0.023 | 1.943 [0.550-6.862] | 0.302 |
| **Age** | 0.876 [0.762-1.008] | 0.065 | 0.995 [0.943-1.050] | 0.854 |
| **BMI** | 1.136 [0.863-1.497] | 0.363 | 0.987 [0.868-1.123] | 0.846 |
| **SUBCUTANEOUS FAT T12 [cm2]** | 0.981 [0.954-1.009] | 0.190 | 0.998 [0.989-1.007] | 0.653 |
| **Sex** | 0.033 [0.002-0.675] | 0.027 | 1.644 [0.449-6.016] | 0.453 |
| **Age** | 0.906 [0.797-1.029] | 0.127 | 0.988 [0.938-1.041] | 0.655 |
| **BMI** | 1.101 [0.836-1.450] | 0.493 | 1.032 [0.906-1.175] | 0.638 |
| **VISCERAL FAT AREA L3 [cm2]** | 1.005 [0.991-1.020] | 0.500 | 0.997 [0.990-1.005] | 0.495 |
| **Sex** | 0.070 [0.005-1.094] | 0.058 | 2.218 [0.576-8.536] | 0.247 |
| **Age** | 0.908 [0.797-1.034] | 0.144 | 0.994 [0.946-1.046] | 0.826 |
| **BMI** | 0.882 [0.685-1.136] | 0.332 | 1.040 [0.921-1.175] | 0.526 |
| **VISCERAL FAT MASS [kg]** | 0.342 [0.070-1.676] | 0.186 | 1.225 [0.752-1.996] | 0.415 |
| **Sex** | 0.025 [0.001-1.008] | 0.050 | 2.219 [0.581-8.478] | 0.244 |
| **Age** | 0.949 [0.842-1.071] | 0.398 | 0.986 [0.936-1.038] | 0.590 |
| **BMI** | 1.006 [0.822-1.232] | 0.951 | 0.978 [0.874-1.094] | 0.692 |
| **WHOLE BODY FAT MASS [kg]** | 0.907 [0.781-1.053] | 0.198 | 1.008 [0.960-1.059] | 0.736 |
| **Sex** | 0.027 [0.001-1.046] | 0.053 | 1.972 [0.518-7.516] | 0.320 |
| **Age** | 0.967 [0.848-1.103] | 0.619 | 0.987 [0.935-1.042] | 0.640 |
| **BMI** | 0.982 [0.811-1.190] | 0.855 | 0.999 [0.907-1.100] | 0.984 |
| **FMR** | 0.213 [0.035-1.276] | 0.090 | 1.492 [0.567-3.931] | 0.418 |
| **Sex** | 0.068 [0.004-1.163] | 0.063 | 1.651 [0.486-5.606] | 0.422 |
| **Age** | 0.869 [0.737-1.026] | 0.098 | 0.982 [0.931- 1.035] | 0.499 |
| **BMI** | 1.040 [0.816-1.325] | 0.752 | 0.969 [0.856-1.097] | 0.620 |

*Abbreviations: IMV = invasive mechanical ventilation, SMA = skeletal muscle area, L3 = at the level of the third lumbar vertebra, SMI = skeletal muscle index, T12 = at the level of the twelfth thoracic vertebra, MRA = muscle radiodensity attenuation, HU = Hounsfield units, FMR = fat to muscle ratio, BMI = body mass index, adj. OR = adjusted odds ratio, CI = confidence interval, Adj. HR = adjusted hazard ratio*
